# A Novel OsteomiRs Expression Signature for Osteoblast Differentiation of Human Amniotic Membrane-Derived Mesenchymal Stem Cells

**DOI:** 10.1155/2019/8987268

**Published:** 2019-03-24

**Authors:** Mariana Avendaño-Félix, Lizeth Fuentes-Mera, Rosalío Ramos-Payan, Maribel Aguilar-Medina, Vanessa Pérez-Silos, Nidia Moncada-Saucedo, Laurence A. Marchat, Juan Antonio González-Barrios, Erika Ruiz-García, Horacio Astudillo-de la Vega, José L. Cruz-Colin, César López-Camarillo

**Affiliations:** ^1^Facultad de Ciencias Químico Biológicas, Universidad Autónoma de Sinaloa, Culiacán Sinaloa, Mexico; ^2^Departamento de Bioquímica y Medicina Molecular, Facultad de Medicina, Universidad Autónoma de Nuevo León, Monterrey, NL, Mexico; ^3^Programa en Biomedicina Molecular y Red de Biotecnología, Escuela Nacional de Medicina y Homeopatía, Instituto Politécnico Nacional, Ciudad de México, Mexico; ^4^Laboratorio de Medicina Genómica, Hospital Regional 1 de Octubre ISSSTE, Ciudad de México, Mexico; ^5^Laboratorio de Medicina Traslacional, Instituto Nacional de Cancerología, Ciudad de México, Mexico; ^6^Laboratorio de Investigación Traslacional en Cáncer y Terapia Celular, Hospital de Oncología, Centro Médico Nacional Siglo XXI, Ciudad de México, Mexico; ^7^Subdirección de Investigación Básica, Instituto Nacional de Medicina Genómica, Ciudad de México, Mexico; ^8^Posgrado en Ciencias Genómicas, Universidad Autónoma de la Ciudad de México, Ciudad de México, Mexico

## Abstract

Human amniotic membrane-derived mesenchymal stem cells (hAM-MSCs) are a potential source of cells for therapeutic applications in bone regeneration. Recent evidence reveals a role for microRNAs (miRNAs) in the fine-tuning regulation of osteogenesis (osteomiRs) suggesting that they can be potential targets for skeleton diseases treatment. However, the functions of osteomiRs during differentiation of hAM-MSCs to osteogenic lineage are poorly understood. In this investigation, we discovered a novel miRNAs expression signature corresponding to the matrix maturation (preosteoblast) and mineralization (mature osteoblast) stages of dexamethasone-induced osteoblastic differentiation of hAM-MSCs. Comprehensive miRNAs profiling using TaqMan Low Density Arrays showed that 18 miRNAs were significantly downregulated, whereas 3 were upregulated in the matrix maturation stage (7 days after osteogenic induction) in comparison to undifferentiated cells used as control. Likewise, 47 miRNAs were suppressed and 25 were overexpressed at mineralization stage (14 days after osteogenic induction) in comparison to osteoprogenitors cells. Five out 93 miRNAs (miR-19b-3p, miR-335-3p, miR-197-3p, miR-34b-39, and miR-576-3p) were regulated at both 7 and 14 days suggesting a role in coordinated guidance of osteoblastic differentiation. Exhaustive bioinformatic predictions showed that the set of modulated miRNAs may target multiple genes involved in regulatory networks driving osteogenesis including key members of BMP, TGF-*β*, and WNT/*β*-catenin signaling pathways. Of these miRNAs, we selected miR-204, a noncoding small RNA that was expressed at matrix maturation phase and downregulated at maturation stage, for further functional studies. Interestingly, gain-of-function analysis showed that restoration of miR-204 using RNA mimics at the onset of mineralization stage dramatically inhibited deposition of calcium and osteogenic maturation of hAM-MSCs. Moreover* in silico* analysis detected a conserved miR-204 binding site at the 3′UTR of TGF-*β*R2 receptor gene. Using luciferase assays we confirmed that TGF-*β*R2 is a downstream effector of miR-204. In conclusion, we have identified a miRNAs signature for osteoblast differentiation of hAM-MSCs. The results from this study suggested that these miRNAs may act as potential inhibitors or activators of osteogenesis. Our findings also points towards the idea that miR-204/TGF-*β*R2 axis has a regulatory role in differentiation of hAM-MSCs committed to osteoblastic lineage.

## 1. Introduction

Osteoporosis disease and bone fractures are among the major public health concerns worldwide, as they produce a decline in patient mobility and a significant increase in medical care costs [1]. A recent review on this subject summarize the evidence indicating that both skeletal development and bone regeneration depend on the proper functional differentiation of mesenchymal stem cells to osteoblasts [2]. Human mesenchymal stem cells are pluripotent stem cells that undergo a multistage differentiation process in which they proliferate and differentiate into osteocytes, adipocytes, and chondrocytes [3]. From the past decade, human amniotic membrane-derived mesenchymal stem cells (hAM-MSCs) have been used for* in vivo* construction of tissue-engineered cartilage, bone, and other soft tissues, and they have become a promising tool for the treatment of bone diseases [4]. Osteoblastic differentiation from hMSCs is a highly specialized process involving the coordinated activation of WNT/*β*-catenin, FGF, and BMPs/TGF-*β* signaling pathways [5, 6], which activate runt-related transcription factor 2 (RUNX2) and Osterix (OSX/Sp7) transcription factors, among others, that in turn activate a complex transcriptional program driving the differentiation of osteoprogenitors towards functional mature osteocytes [7]. Therefore, elucidating the molecular mechanisms that regulate the osteoblastic differentiation is essential to help understand the molecular mechanisms of osteogenesis, but it also may be a guide for the development of novel therapies for the treatment of bone lesions and osteoporosis.

MicroRNAs (miRNAs) are evolutionary conserved single-stranded small RNA molecules of 21-25 nucleotides in length that function as negative regulators of gene expression [8]. MiRNAs function as guide molecules in posttranscriptional gene silencing acting by complementary binding with the 3′ untranslated region (UTR) of specific transcripts leading to target mRNA degradation and/or translational repression in P-bodies [9]. Recent evidences suggests an important role for miRNAs in the fine-tuning regulation of genes involved in osteogenesis indicating that these so-called osteomiRs can be promising therapeutic targets for skeleton diseases [10–12]. However, the functions of miRNAs during differentiation of hAM-MSCs to osteogenic lineage are still poorly understood. Despite this interest, comprehensive miRNAs profiling studies associated with differentiation of hAM-MSCs to osteoblasts are scarce; thus the relevance of small RNAs in osteogenesis is not yet known. In this investigation, we reported a novel miRNAs expression signature in two stages of hAM-MSCs differentiation to osteoblasts and functionally characterized the role of miR-204 in this cellular process. Implications on the molecular mechanisms regulating the differentiation of hAM-MSCs and the potential therapeutic applications are discussed.

## 2. Materials and Methods

### 2.1. Isolation of Stem Cells from the Human Amniotic Membranes

Caesarean-delivered term placentas (n=4) were collected from healthy donor mothers. Procedures were approved by the Ethics Committee of the University Hospital “Dr. José Eleuterio González”, and each donor gave her consent. Pieces of amniotic membrane (10 x10 cm) were subjected to two enzymatic digestions by adding (i) 0.125% trypsin/0.5 mM EDTA solution at 37°C for 30 min and (ii) 100 U/mL collagenase type II and 3 mM calcium chloride in the Dulbecco's Modified Eagle's Medium (DMEM) for 2 h at 37°C, followed by washing with PBS. The resulting cell suspension was filtered, and cells were seeded in 25-cm^2^ flasks in DMEM containing 10% fetal bovine serum (FBS) and 100 U/mL penicillin, 100 mg/mL streptomycin, and 0.25 mg/mL amphotericin B. The resulting hAM-MSCs were cultured in a humidified 5% CO_2_ atmosphere at 37°C until 90% confluence. All experiments were carried out using hAM-MSCs below 10 passages.

### 2.2. Fluorescent Activated Cell Sorting

After establishing the hAM-MSCs population, a CD44^+^/CD73^+^/CD105^−^ (CD105^−^) subpopulation was enriched by Fluorescent Activated Cell Sorting (FACS Aria, BD). hAM-MSCs were stained with human PE-conjugated CD73, APC-conjugated CD105 and PercP-Cy5.5-conjugated CD44 (Miltenyi Biotec), and positive cells were collected. After centrifugation at 400 g for 5 min, they were plated in fresh culture medium for expansion and further molecular characterization.

### 2.3. Osteogenic Differentiation of hAM-MSCs

The osteogenic differentiation was induced as previously described [13]. Briefly, isolates of hAM-MSCs (2x10^3^ cells/cm^2^) were grown in osteogenic medium (DMEM supplemented with 10% FBS, 10 mM *β*-glycerophosphate, 0.25 mM ascorbic acid, and 10^-8 ^M dexamethasone). Cultures were maintained with medium changes every 2-3 days. Cells were stained with Alizarin Red S dye reactive (pH 4.3) to evaluate calcium-rich deposits and monitor the differentiation process as described [14]. Cells were imaged and birefringence staining was quantified using the FluorChem 9900 system.

### 2.4. Western Blot Assays

For immunoblotting, hAM-MSCs were rinsed with cooled PBS and lysed at room temperature for 10 min in 1 mL of RIPA buffer (20 mM Tris-HCl, pH 7.5, 150 mM NaCl, 1 mM EGTA, 1% NP-40, 1% sodium deoxycholate, 2.5 mM sodium pyrophosphate, 1 mM b-glycerophosphate, 1 mM Na_3_VO_4_, and 1 mg/mL leupeptin) containing the Complete Protease Inhibitor (0.5 mM phenylmethylsulfonyl fluoride, 10 mg/mL leupeptin, 10 mg/mL aprotinin, 5 mg/mL pepstatin, 10 mg/mL soybean trypsin inhibitor, and 0.5 mM dithiothreitol; ROCHE, Molecular Biochemicals). Equal amounts of protein (30 *μ*g) were run on 10% SDS-PAGE gels and transferred to a PVDF membrane (Millipore Corporation). The membrane was blocked for 60 min at room temperature with TBST-1 (137 mM NaCl, 20 mM Tris, and 0.1% Tween-20 (pH 7.6)) containing 5% BSA (Sigma-Aldrich) and then incubated overnight at 4°C with rabbit anti-human COL1A2 (1:1,000) antibodies. The membrane was washed in TBST-1 and incubated with horseradish peroxidase-conjugated goat anti-rabbit IgG antibody (1:3000, Zymed). Antibody-antigen complexes were revealed using the ChemiLucent system (Chemicon) and imaged. Densitometry analysis was performed using the myImage Analysis software.

### 2.5. MicroRNAs Profiling Using TaqMan Low Density Arrays

Total RNA (70 ng) was isolated from undifferentiated hAM-MSCs (time 0), and cells collected after 7 and 14 days of osteogenic induction using the TRIzol reagent (Invitrogen). MiRNAs expression profiles were obtained using the stem-loop qRT-PCR-based TaqMan Low Density Arrays (TLDAs) covering 677 human mature miRNAs (https://www.thermofisher.com/order/catalog/product/4398965). Briefly, expression of miRNAs was performed by reverse transcription and quantitative real-time polymerase chain reaction using the Megaplex TaqMan Low Density Arrays v2.0 system 4398965 (Applied Biosystems. Foster City, CA) as described by the manufacturer. In order to detect low abundant miRNAs a preamplification step was included. For this, 12.5 *μ*l TaqMan PreAmp Master Mix was incubated with 2.5 *μ*l Megaplex PreAmp Primers and water up to 22.5 *μ*l. Then, 2.5 *μ*l of RT reaction was added to the PreAmp mix and sequences were amplified following the next program: 95°C for 10 min (enzyme activation), 55°C for 2 min (annealing), 72°C for 2 min (extension), 95°C for15 s (denaturation), 69°C for 4 min (annealing/extension), and 99°C for 10 min (enzyme inactivation). Then, preamplified products were loaded into the TLDAs for PCR reactions and amplification signal detection was performed in the 7900 FAST real-time thermal cycler (ABI). Tests were normalized using RNU44 as control.

### 2.6. Reverse Transcription and Real-Time Polymerase Chain Reaction

Quantitative real-time RT-PCR (qRT-PCR) analysis for miRNAs expression was performed using the TaqMan MicroRNA Assay kits (ThermoFisher). Total RNA (100 ng) was reverse transcribed using a looped RT specific primer, dNTPs (100 mM), reverse transcriptase MultiScribe (50 U/*μ*l), 10X buffer, RNase inhibitor (20 U/*μ*l), and 4.16 *μ*l RNase-free water. Retrotranscription reaction (1:15) was mixed with master mix TaqMan (Universal PCR Master Mix, No AmpErase UNG, 2X), and the corresponding specific TaqMan PCR probe. PCR reaction was performed in a GeneAmp System 9700 (Applied Biosystems) as follows: 95°C for 10 min and 40 cycles at 95°C for 15 s and 60°C for 1 min. Tests were normalized using RNU44 as endogenous control.

### 2.7. Prediction of Gene Targets and Gene Ontology Analysis

Target genes of miRNAs were predicted using TargetScan, PicTar5, miRanda, and DIANA microT software. Only targets predicted by two algorithms were included in further analyses. Cellular pathways and processes potentially affected by let-7c-3p were predicted using DAVID 6.7 software.

### 2.8. Transfection of miR-204 Mimics

Seven days after induction of osteogenic differentiation, hAM-MSCs were transfected with miR-204 mimics (40 nM) and scramble (40 nM) sequence (AM17110, ThermoFisher) as negative control using siPORT amine transfection agent (Ambion). Briefly, miR-204 mimics was diluted in 25 *μ*l of Opti-MEM to 40 nM concentrations and then added to wells containing cultured cells in 450 *μ*l of DMEM. After 48 h incubation, total RNA was extracted using TRIzol and efficacy of miR-204 restoration was evaluated by qRT-PCR using specific stem-looped RT oligonucleotide and TaqMan probe as implemented in the TaqMan MicroRNA assays protocol.

### 2.9. Effects of miR-204 Mimics on Osteogenic Differentiation of hAM-MSCc

Briefly, 11 days after of osteoblastic differentiation the cells were transfected with 30 nM of miR-204 mimics using siPORT amine transfection agent (Ambion), and 48 h after transfection the calcium-rich deposits were evaluated with Alizarin Red S dye reactive (pH 4.3) which binds to intracellular calcium ions. Cells were incubated with dye for 15 min at room temperature and washed tree times with PBS 1x, and then cells were imaged and birefringent staining was quantified using FluorChem 9900 for comparisons of pixel intensity between control nontransfected and miR-204 mimics transfected hAM-MSCs.

### 2.10. Luciferase Gene Reporter Assays

A DNA fragment of the 3′UTR of TGF-*β*R2 gene containing the predicted miR-204 binding site and as well as a mutated version of the seed region were cloned into the p-miR-report vector (Ambion) downstream of the luciferase gene. Constructs were verified through automatic sequencing. Then, recombinant pmiR-LUC- TGF*β*R2 wild type and pmiR-LUC-TGF-*β*R2 mutant plasmids were transfected into hAM-MSCs cells using lipofectamine 2000 (Invitrogen). At 24 h after transfection, cells were cotransfected with miR-204 mimics and scramble (40 nM) and incubated for 24 h. Finally, firefly and* Renilla reniformis* luciferase activities were measured by the Dual-Glo luciferase Assay (Promega) using a Fluoroskan Ascent™ Microplate Fluorometer, and firefly luciferase activity was normalized with Renilla reniformis luciferase data.

### 2.11. Statistical Analyses

Experiments were performed three times by triplicate and results were represented as mean ± SD. One-way analysis of variance (ANOVA) was used to compare the differences between means. A p<0.05 was considered as statistically significant.

## 3. Results

### 3.1. Osteoblastic Differentiation of hAM-MCS In Vitro

**Figure 1:**
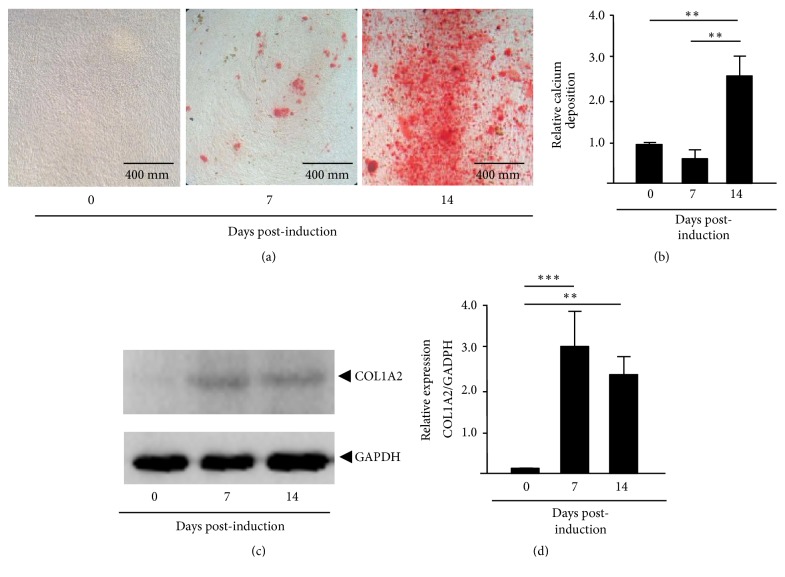
*Osteogenic differentiation of AM-hMSCs.* (a) Alizarin Red staining showing mineral deposition of calcium in undifferentiated AM-hMSCs (time 0) and after 7 and 14 days of differentiation to osteoblastic lineage. (b) Quantification of the calcium deposition shown in (a). (c) Western blot assays using whole protein extract and antibodies raised against COL1A2 protein after 0, 7, and 14 days of induction. Data were normalized using control GAPDH expression. (d) Densitometry analysis of the immunodetected bands in (c). Data represent the mean ± SD of three independent experiments (*∗∗p*<0.05; *∗∗∗p*<0.001).

Previously, we have isolated and characterized the biological events underlying the osteoblastic differentiation of the hAM-MSCs subpopulation studied here and established the temporality of proliferation (undifferentiated cells, time 0), matrix maturation (preosteoblast, 7 days), and mineralization (mature osteoblast, 14 days) stages [13, 14]. To assess the ability of hAM-MSCs isolates to differentiate into osteogenic lineage, the calcium deposition and expression of the mineralization stage marker collagen type I-alpha (COL1A2) were measured. The hAM-MSCs were induced to differentiation in osteogenic media and maintained for three weeks at 37°C. Cultures were stained with Alizarin Red at 0, 7, and 14 days after induction and imaged. Results showed a gradual and significant time-dependent increase of calcium-rich deposits in preosteoblasts and osteoblasts in comparison to undifferentiated control cells (time 0) (Figures 1(a) and 1(b)). Congruently, immunoblot assays revealed that the COL1A2 protein expression was significantly increased at 7 and 14 days after induction relative to undifferentiated control cells (Figures 1(c) and 1(d)). These data confirmed that the isolates of hAM-MSCs used in this study efficiently initiated the process of differentiation to osteoblasts as previously described [13, 14].

### 3.2. MicroRNAs Expression Signatures of hAM-MSCs Differentiation to Osteoblastic Lineage

**Figure 2:**
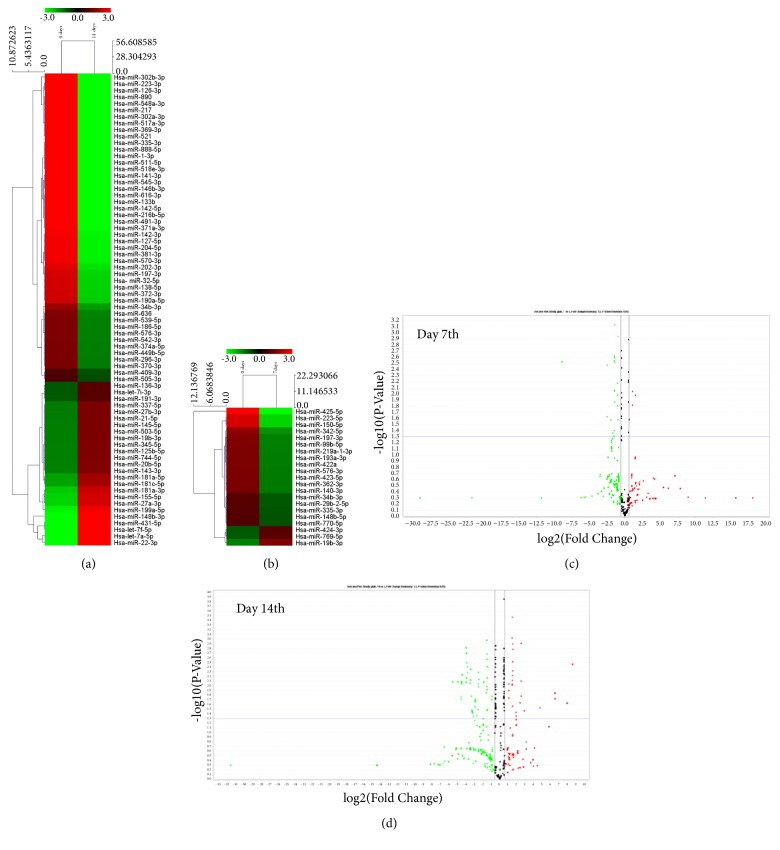
*Expression profiling of miRNAs modulated during osteoblastic differentiation of hAM-hMSCs.* (a) Unsupervised hierarchical clustering analysis displaying the differential expression of miRNAs at 14 days and (b) 7 days of osteoblastic differentiation relative to time 0 (undifferentiated hAM-MSCs). The heatmap (Spearman correlation; Euclidean distance) represents a cluster analysis of the logarithm of transformed ΔΔCt values of the differentially expressed microRNAs. Color key: upregulated miRNAs (red); downregulated miRNAs (green). (c and d) Volcano plot representations of miRNAs modulated at 7 and 14 days. The y-axis represents the mean expression value of log10 (P-value) and the x-axis displays the log2-fold change value. Upregulated and downregulated miRNAs are shown in red and green, respectively. Black dots indicate genes with no significant change in expression.

**Figure 3:**
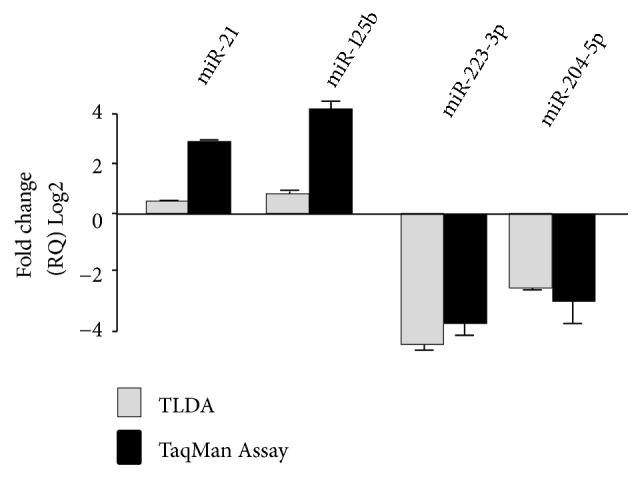
*Quantitative RT-PCR assays*. Validation of expression levels of four miRNAs modulated after 14 days of differentiation to osteoblastic lineage using qRT-PCR TaqMan assays (black bars) in comparison with data obtained from TLDA (grey bars). Data were expressed as mean ± SD.

To evaluate the potential contribution of miRNAs in osteoblast maturation of hAM-MSCs, we comprehensively analyzed 667 miRNAs using stem-loop qRT-PCR in TaqMan Low Density Arrays (TLDAs) as previously described [15]. Undifferentiated hAM-MSCs were induced to osteoblastic lineage in differentiation media and global miRNAs profiles were assessed at matrix maturation (7 days after osteogenic induction) and mineralization (14 day after osteogenic induction) stages. After comparative 2-ΔΔCt analyses, a total of 93 miRNAs significantly modulated (|log⁡2(T/N)| > 1.0;* p*<0.05) were identified. Of these, 18 miRNAs showed a significant downregulation, whereas three were upregulated at matrix maturation stage in comparison to undifferentiated cells (Table 1). Likewise, 47 miRNAs were significantly suppressed and 25 were overexpressed at mineralization stage (Table 2). This panel of up- and downregulated miRNAs was able to separate the preosteoblast and mature osteoblast groups relative to undifferentiated cells as depicted in the 2-way unsupervised hierarchical clustering shown in Figures 2(a) and 2(b). A volcano plot representation of miRNAs deregulated at 7 and 14 days is shown for easy visualization in Figures 2(c) and 2(d), respectively. Interestingly, five out 93 miRNAs were modulated at both times: miR-19b-3p was upregulated, whereas miR-335-3p, miR-197-3p, miR-34b-3p, and miR-576-3p were downregulated, suggesting an important role in the coordinated guidance of the osteoblastic differentiation process. Then, we proceeded to validate the changes in expression of four selected miRNAs using specific probes in qRT-PCR assays. Representative data at 14 days after induction showed that levels of miR-21, miR-125b, miR-204, and miR-123 detected by qRT-PCR were similar to those found in TLDAs platform (Figure 3).

### 3.3. Genes and Signaling Pathways Potentially Impacted by Regulated OsteomiRs

To gain a comprehensive understanding about the impact of miRNAs abundance changes during the osteogenic differentiation of hAM-MSCs, we performed literature searches for reports on the set of regulated miRNAs. Results indicated that a number of miRNAs have well-known functions in osteogenesis. For instance, five osteomiRs (miR-223-5p, miR-193a-3p, miR-29b-2-5p, miR-335-3p, and miR-148b-5p) with a significant repression at 7 days of differentiation have been related to osteogenesis (Table 1). Notably, previous studies showed that restoration of miR-223-5p impaired the osteogenic differentiation of ST2 mesenchymal stem cells by targeting FGFR2 [16]. Likewise, nine of overexpressed miRNAs (miR-119a-5p, miR-21-5p, miR-145-5p, miR-503-5p, miR-125b-5p, miR-181a-5p, miR-155-5p, let-7f-5p, and miR-22-3p) at 14 days have been reported in osteogenesis (Table 2). For example, miR-119a-5p was found upregulated during osteogenic differentiation of hMSCs; and nanoparticle delivery of miR-119a-5p resulted in enhanced maturation of hMSCs* in vitro* [17]. Importantly, most of miRNAs detected here as modulated have not been previously involved in osteoblastic differentiation, suggesting that they may represent novel miRNAs with potential therapeutic applications.

To understand the biological impact of miRNAs variations during early and late stages of osteogenesis, we computationally predicted their gene targets using a combination of TargetScan, PicTar5, miRanda, and DIANA microT prediction tools. Exhaustive bioinformatic analysis yielded a large list of putative gene targets. In order to associate miRNAs function to specific cellular pathways and processes, the complete list of putative gene targets was filtered using the Gene Ontology (GO) terms function as implemented in DAVID databases. In Tables 3 and 4, we present an overview of the main signaling pathways impacted by the complete dataset of miRNAs regulated at both 7 and 14 days after osteogenic differentiation. For instance, predictions showed that 17 miRNAs modulated at 7 days may be targeting at least 42 different genes involved in the osteogenesis-related TGF-*β* signaling pathway. In addition, 20 miRNAs were predicted to target 65 genes involved in signaling pathways regulating pluripotency of stem cells (Table 3). These data confirmed that an important number of modulated miRNAs are related to known cellular pathways associated with hAM-MSCs osteogenic differentiation.

### 3.4. Overexpressed OsteomiRs Target Multiple Genes Involved in TGF-*β* and WNT/*β*-Catenin Pathways

**Figure 4:**
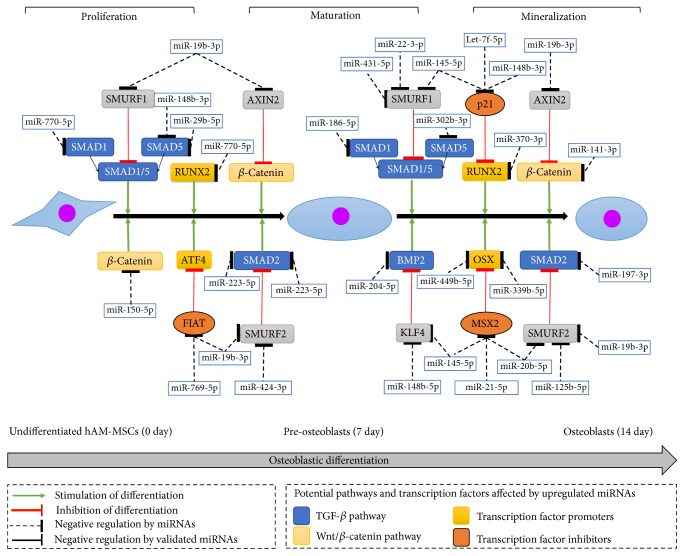
*Model for regulation of osteogenesis by miRNAs impacting TGF-β and WNT/β-catenin pathways.* The schema shows a subset of modulated miRNAs at 7 (maturation) and 14 (mineralization) days after the dexamethasone-induced differentiation of AM-hMSCs to osteoblasts relative to control undifferentiated cells (time 0). Selected miRNAs and their predicted targets involved in WNT/*β*-catenin and TGF*β* signaling pathways are depicted. Proteins that stimulate and repress the osteogenic differentiation process are denoted with green and red lines, respectively. Molecular functions of gene targets and corresponding pathways are denoted in colors.

In order to better characterize the impact of modulated miRNAs on the osteogenesis process, we next focused on the set of upregulated miRNAs and found that they may attenuate a large list of key genes involved in TGF-*β* and WNT/*β*-catenin pathways which are critical for osteogenic differentiation and bone formation. Several of these overexpressed miRNAs may enhance or impair the osteoblasts differentiation by targeting their cognate activator or repressor genes involved in TGF-*β* signaling, such as RUNX2, B-catenin, ATF4, and OSX transcription factors which act as activators of osteogenic differentiation, as well as FIAT, p21, SMURF, and MSX2 which function as repressors (Figure 4). For instance, miR-19b-3p may stimulate differentiation by targeting FIAT which is a repressor of the osteogenesis-activator ATF4 (Figure 4). Likewise, at both early and late times of osteogenic differentiation, miR-19b-3p may also enhance osteogenesis by targeting AXIN2 which is a repressor of *β*-catenin. A more complex regulation may be occurring for some genes. At late stages of osteogenic differentiation, let-7f-5p, miR-145-5p, and miR-148b-3p were predicted to cooperatively target p21, a repressor of RUNX2, which could stimulate osteoblasts mineralization. In contrast, miR-150-5p could inhibit osteoblastic differentiation by targeting *β*-catenin in undifferentiated cells. Similarly, at late stage of maturation, the dual targeting of Osteryx (OST) by miR-449b-5p and miR-339b-5p could result in osteogenesis impairment (Figure 4).

**Figure 5:**
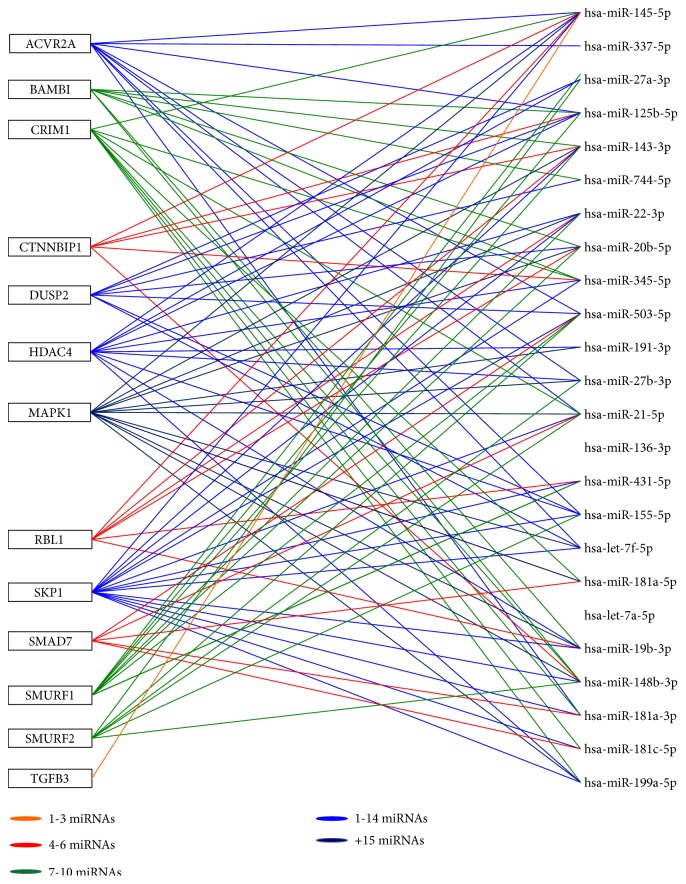
*Representative miRNA/mRNA interaction network for osteogenic differentiation of hAM-MSCs at mineralization stage*. The pairs miRNAs/mRNA in the interaction network were generated based on the expression levels of upregulated miRNAs at mineralization stage (14 days) and their predicted gene targets with known inhibitory functions in the TGF-*β* pathway. The solid color lines connecting molecules represent miRNA-mRNA interaction. The interaction network showed deregulated subnetworks that are clearly separate depending on the number of targets for each miRNA, for instance, genes potentially modulated by 1-3 upregulated miRNAs (in orange), and others by 4-6 miRNAs (in green).

In addition, we found that a number of target genes could be recognized by more than one miRNA indicating an extensive target gene redundancy and at the same time revealing complex and intricate miRNA/mRNA regulation networks. As an example, in Figure 5 we depicted a miRNA/mRNA interactions network for miRNAs upregulated at 14 days and their predicted targets with known inhibitory functions in TGF-*β* and WNT/*β*-catenin pathways.

### 3.5. MiR-204 Inhibits the Osteoblast Differentiation by Targeting TGF-*β*R2

**Figure 6:**
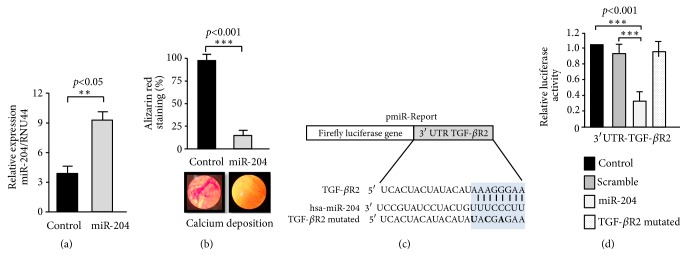
*MiR-204 inhibits the osteogenic differentiation of AM-hMSCs by targeting TGF-βR2*. (a) Relative expression of miR-204 after transfection of RNA mimics in AM-hMSCs at 13 days after dexamethasone-induced osteoblastic differentiation. (b) Quantification of Alizarin Red staining for calcium deposition (bottom images) in nontransfected AM-hMSCs (control) or transfected cells with miR-204 mimics. (c) Schematic representation of p-miR report construct containing the 3′UTR of TGF-*β*R2 gene cloned downstream of the firefly luciferase gene. Seed sequence is indicated in blue shaded box. Point mutations in the miR-204 binding site of 3′UTR of TGF-*β*R2 gene are denoted in bold. (d) Luciferase assays in AM-hMSCs cotransfected with miR-204 mimics and the constructs described in (c). Nontransfected and scramble transfected cells were used as controls. Data represent the mean ± SD of three independent experiments (*∗∗∗p*<0.001; *∗∗p*<0.05).

To initiate the study of the molecular mechanisms underlying the regulation of hAM-MSCs differentiation, we focused on miR-204, a noncoding small RNA overexpressed at the matrix maturation stage and downregulated at the maturation stage. Bioinformatics predictions showed that miR-204 may target multiple key genes involved in osteogenesis promotion including ACVR1C, ACVR2B, TGBR2, BMPR1A, BMPR2, SMAD2, and SMAD5 among other; thus it could have a relevant role in osteoblast differentiation. To evaluate the role of miR-204 in osteogenic differentiation of hAM-MSCs, we first restored its expression using RNA mimics at the onset of mineralization stage (Figure 6(a)). Data showed that miR-204 inhibited deposition of calcium and osteogenic maturation of hAM-MSCs (Figure 6(b)). Then, we searched for potential downstream targets of miR-204 using different miRNA target prediction tools.* In silico* analysis detected a conserved miR-204 binding site at the 3′UTR of TGF-*β*R2 receptor gene. Thus it was reliable to propose that restoration of miR-204 levels may negatively regulate osteoblast differentiation through direct targeting of TGF-*β* signaling. To corroborate this hypothesis, we performed luciferase reporter gene assays. A DNA fragment of the 3′UTR containing the TGF-*β*R2 binding site for miR204 was cloned downstream of the luciferase gene into the pmiR report vector (Figure 6(d)). In addition, point mutations in the predicted miR-204 binding site of the 3′UTR were included in the analysis. Recombinant pmiR-LUC-TGF-*β*R2 plasmid was transfected during differentiation of hAM-MSCs and luciferase activity was analyzed after 24 h of transfection. Data showed that ectopic expression of miR-204 and cotransfection of pmiR-LUC-TGF-*β*R2 construct resulted in a significantly reduction of the relative luciferase activity in comparison with controls (Figure 6(d)). When mutated sequences of the 3′UTR TGF-*β*R2 were assayed, no significant changes in luciferase activity were found, indicating that miR-204 binding was specific.

## 4. Discussion

Human MSCs were initially isolated from bone marrow [18], and they have been later identified in several fetal tissues such as the liver, bone marrow, and pancreas, in the endothelial umbilical vein and in the preterm blood of the fetus [19]. The International Society of Cellular Therapy (ISCT) has described MSCs as cells that express the surface markers CD73, CD105, CD90, and CD44 in the absence of hematopoietic stem cell markers and differentiate into mesenchymal lineages such as osteoblasts, adipocytes, and chondroblasts* in vitro* [4]. Several miRNAs modulate osteogenic differentiation; thus they have been dubbed as osteomiRs. For instance, miR-125b regulates osteoblastic differentiation of human MSCs through targeting of BMPR1b [20], whereas miR-223 suppresses osteoblast differentiation by inhibiting DHRS3 [21]. However, miRNAs functions during differentiation of hAM-MSCs to osteogenic lineage have not been completely explored.

In order to contribute to the understanding of miRNAs roles in osteogenic differentiation of hAM-MSCs, here we performed a miRNAs profiling in MSCs obtained from human placenta. Our findings indicated a novel miRNAs expression signature corresponding to preosteoblast and mature osteoblast stages of dexamethasone-induced osteoblastic differentiation of hAM-MSCs. Bioinformatic predictions showed that these miRNAs may target multiple genes involved in regulatory networks driving osteogenesis including members of BMP, TGF-*β*, and WNT/*β*-catenin signaling pathways. Importantly, our results confirmed that a number of miRNAs have well-known functions in osteogenesis. For instance at 7 days of differentiation, we found a significant repression of miR-29b relative to osteo-progenitors cells (Table 1). Likewise, ectopic expression of miR-29b resulted in low levels of osteogenesis inhibitors CDK6 and HDAC4 and stimulated the osteogenic differentiation of unrestricted somatic stem cells from human cord blood [22]. At 14 days, we identified that miR-119a-5p and miR-22-3p previously reported in osteogenesis are overexpressed in hAM-MSCs (Table 2). Similarly, it was described that upregulation of miR-22 promotes the osteogenic differentiation of human adipose tissue-derived mesenchymal stem cells by repressing the osteogenesis repressor HDAC6 [23]. These data suggested that miRNAs detected here as regulated could be regulating the differentiation of hAM-MSCs, importantly; most of miRNAs detected here as modulated have not been previously connected to osteoblastic differentiation, suggesting that they may represent novel miRNAs with potential therapeutic applications.

In addition, we found that the set of upregulated miRNAs may regulate a large list of key genes involved in TGF-*β* and WNT/*β*-catenin pathways, which are critical for osteogenic differentiation and bone formation. This panel of deregulated miRNAs represents a guide for further functional characterization of the osteogenesis process. To go beyond the miRNAs profiling, we initiated the functional analysis of one deregulated miRNAs related to osteogenesis. Our data indicated that restoration of miR-204, a noncoding small RNA that was expressed at matrix maturation stage and downregulated in maturation stage, impaired differentiation at least by targeting TGF-*β*R2 which is regulator of RUNX2 gene, the master regulator of stem cells differentiation.

## 5. Conclusions

In summary, here we reported a novel miRNAs signature during differentiation of hAM-MSCs to osteogenic lineage which may target multiple genes involved in regulatory networks driving osteogenesis including members of BMP, TGF-*β*, and WNT/*β*-catenin signaling pathways. Our findings suggested an unexpected regulatory role for the miR-204/TGF-*β*R2 axis in differentiation of hAM-MSCs committed to osteoblastic lineage. These data may serve as a guide for further functional analysis focused in the implementation of osteomiRs as novel therapeutic tools in bone diseases.

## Figures and Tables

**Table 1:** MicroRNAs expression profile of hAMSC after 7 days of differentiation to osteoblasts.

Down-regulated	Fold change (log2)	*p-*value	Chromosomal location
miR-425-5p	-8.90	0.003	3p21.31
**miR-223-5p**	-2.53	0.023	Xq12
miR-150-5p	-2.52	0.003	19q13.33
miR-342-5p	-1.73	0.043	14q32.2
Hsa-miR-197-3p	-1.52	0.039	1p13.3
miR-99b-5p	-1.51	0.003	19q13.41
miR-219a-1-3p	-1.50	0.002	6p21.32
**miR-193a-3p**	-1.48	0.009	17q11.2
miR-422a	-1.48	0.009	15q22.31
miR-576-3p	-1.47	0.014	4q25
miR-423-5p	-1.46	0.003	17q11.2
miR-362-3p	-1.44	0.003	Xp11.23
miR-140-3p	-1.44	0.001	16q22.1
miR-34b-3p	-1.04	0.015	7q32.2
**miR-29b-2-5p**	-1.04	0.007	1q32.2
**miR-335-3p**	-1.01	0.010	5q33.3
**miR-148b-5p**	-0.99	0.001	7p15.2
mir-770-5p	-0.98	0.026	14q32.2

Up-regulated	Fold change (log2)	*p-*value	Chromosomal location

miR-424-3p	1.05	0.015	Xq26.3
miR-769-5p	1.06	0.009	19q13.32
miR-19b-3p	1.51	0.010	13q31.3

miRNAs previously related to osteogenesis are denoted in bold.

**Table 2:** MicroRNAs expression profile of hAM-MSCs after 14 days of differentiation to osteoblasts.

Down-regulated	Fold change (log2)	p-value	Chromosomal location

miR-302b-3p	-5.50	0.008	4q25
miR-223-3p	-4.90	0.008	Xq12
**miR-126-3p**	-4.78	0.007	9q34.3
miR-890	-4.53	0.020	Xq27.3
miR-548a-3p	-4.52	0.017	6p22.3
**miR-217**	-4.49	0.009	2p16.1
**miR-302a-3p**	-4.48	0.009	4q25
miR-517a-3p	-4.48	0.009	19q13.42
miR-369-3p	-4.46	0.008	14q32.31
miR-521	-4.41	0.006	19q13.42
miR-335-3p	-3.95	0.008	7q32.2
miR-888-5p	-3.95	0.002	Xq27.3
miR-1-3p	-3.92	0.002	20q13.33
miR-511-5p	-3.82	0.004	10p12.33
miR-518e-3p	-3.79	0.005	19q13.42
**miR-141-3p**	-3.58	0.004	12p13.31
miR-545-3p	-3.47	0.009	Xq13.2
miR-146b-3p	-3.44	0.008	10q24.32
miR-616-3p	-3.33	0.017	12q13.3
**miR-133b**	-3.28	0.033	6p12.2
miR-142-5p	-3.27	0.034	17q22
miR-216b-5p	-3.23	0.036	2p16.1
miR-491-3p	-3.19	0.038	9p21.3
miR-371a-3p	-3.10	0.046	19q13.42
miR-142-3p	-2.91	0.020	17q22
miR-127-5p	-2.90	0.022	14q32.2
**miR-204-5p**	-2.89	0.028	9q21.12
miR-381-3p	-2.89	0.021	14q32.31
miR-570-3p	-2.87	0.020	3q29
miR-202-3p	-2.69	0.043	10q26.3
miR-197-3p	-2.54	0.033	1p13.3
miR-32-5p	-2.50	0.009	9q31.3
**miR-138-5p**	-2.49	0.005	3p21.32
miR-372-3p	-2.47	0.011	19q13.42
miR-190a-5p	-2.45	0.007	15q22.2
miR-34b-3p	-1.88	0.008	11q23.1
miR-636	-1.56	0.014	17q25.1
miR-539-5p	-1.51	0.001	14q32.31
miR-186-5p	-1.50	0.003	1p31.1
miR-576-3p	-1.50	0.002	4q25
miR-542-3p	-1.50	0.011	Xq26.3
miR-374a-5p	-1.49	0.034	Xq13.2
miR-449b-5p	-1.49	0.012	5q11.2
miR-296-3p	-1.48	0.004	20q13.32
miR-370-3p	-1.46	0.032	14q32.31
miR-409-3p	-0.95	0.02	14q32.31
miR-505-3p	-0.90	0.017	Xq27.1

Up-regulated	Fold change (log2)	*p-*value	Chromosomal location

miR-136-3p	1.05	0.013	14q32.2
let-7i-3p	1.05	0.018	12q14.1
miR-191-3p	1.06	0.009	3p21.31
miR-337-5p	1.46	0.001	14q32.2
miR-27b-3p	1.46	0.009	9q22.32
**miR-21-5p**	1.48	0.010	17q23.1
**miR-145-5p**	1.49	0.001	5q32
**miR-503-5p**	1.49	0.008	Xq26.3
miR-19b-3p	1.49	0.027	13q31.3
miR-345-5p	1.50	0.006	14q32.2
**miR-125b-5p**	1.51	0.001	21q21.1
miR-744-5p	1.51	0.003	17p12
miR-20b-5p	1.53	0.005	Xq26.2
miR-143-3p	1.54	0.002	5q32
**miR-181a-5p**	1.91	0.037	9q33.3
miR-181c-5p	1.93	0.044	19p13.12
miR-181a-3p	2.39	0.023	9q33.3
**miR-155-5p**	2.50	0.017	21q21.3
miR-27a-3p	2.51	0.001	19p13.12
**miR-199a-5p**	2.88	0.034	19p13.2
miR-148b-3p	4.73	0.029	12q13.13
miR-431-5p	6.46	0.014	14q32.2
**let-7f-5p**	6.50	0.019	9q22.32
let-7a-5p	7.89	0.024	9q22.32
**miR-22-3p**	8.51	0.003	17p13.3

miRNAs previously related to osteogenesis are denoted in bold.

**Table 3:** Cellular signaling pathways regulated by miRNAs regulated at 7 days of differentiation to osteoblasts.

KEGG pathway	*p-*value	Number of target genes	Number of miRNAs
TGF*β* pathway	0.0002	42	17
PI3K-Akt pathway	0.0005	152	20
Hippo pathway	0.0006	68	20
FoxO pathway	0.0013	68	20
Ubiquitin mediated proteolysis	0.0015	67	18
Ras pathway	0.0020	105	21
ErbB pathway	0.0024	46	19
Rap1 pathway	0.0024	98	21
Phosphatidylinositol signaling system	0.0028	43	16
AMPK pathway	0.0062	60	18
mTOR pathway	0.0078	33	18
Signaling pathways regulating pluripotency of stem cells	0.0099	65	20
Focal adhesion	0.0136	92	20
MAPK pathway	0.0259	111	21
Calcium pathway	0.0322	78	17

**Table 4:** Cellular signaling pathways regulated by miRNAs modulated at 14 days of differentiation to osteoblasts.

KEGG pathway	*p-*value	Number of target genes	Number of miRNAs
ECM-receptor interaction	8.03e-11	63	56
Hippo signaling pathway	2.62e-07	115	64
Focal adhesion	3.11e-07	159	64
ErbB signaling pathway	3.45e-07	72	60
TGF-beta signaling pathway	5.50e-07	63	59
PI3K-Akt signaling pathway	8.54e-07	247	64
Ras signaling pathway	2.29e-05	160	63
Wnt signaling pathway	7.70e-05	105	63
MAPK signaling pathway	0.0002	179	63
Rap1 signaling pathway	0.0002	152	62
Signaling pathways regulating pluripotency of stem cells	0.0004	103	65
Protein processing in endoplasmic reticulum	0.0021	116	59
FoxO signaling pathway	0.0021	96	62
Adherens junction	0.0064	58	55
Ubiquitin mediated proteolysis	0.0123	98	60
mTOR signaling pathway	0.0128	46	58
Gap junction	0.0139	64	60
cGMP-PKG signaling pathway	0.0192	113	63
cAMP signaling pathway	0.0211	134	63
p53 signaling pathway	0.0228	50	55
Notch signaling pathway	0.0430	36	35
AMPK signaling pathway	0.0467	87	61

## Data Availability

All data used to support the findings of this study are included within the article. The raw data obtained from the miRNAs profiling in TLDAs platform used to support the findings of this study are available from the corresponding author upon request.
